# Supplementary material for: Progesterone Receptor Membrane Component 1 Regulates Cellular Stress Responses and Inflammatory Pathways in Chronic Neuroinflammatory Conditions
Source: Antioxidants (Basel). 2024 Feb 13;13(2):230. doi: 10.3390/antiox13020230 (PMC10886208; doi:10.3390/antiox13020230)
Supplement: Supplementary file 1 [file antioxidants-13-00230-s001.zip › antioxidants-2781811-supplementary.pdf]

Supplementary Material

1. Supplementary Figures

(A)

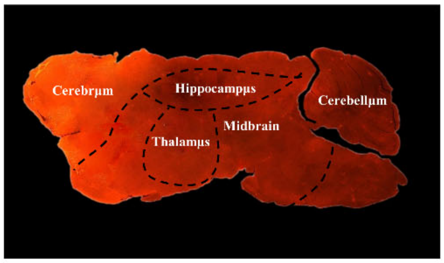

(B)

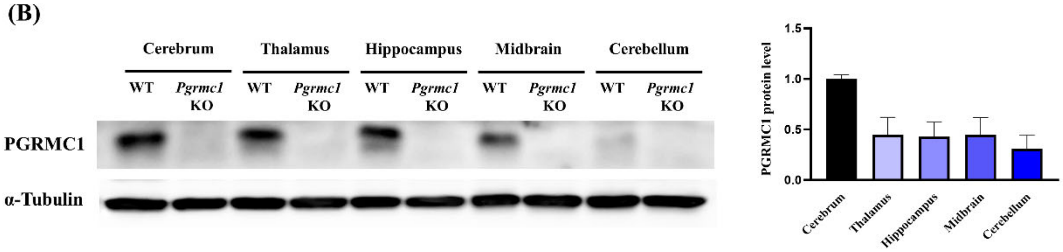

Figure S1. PGRMC1 protein expression in mouse brain. (A) Extracted regions of the brain. (B) Western blot analysis and quantification of PGRMC1 were evaluated in five regions of the brain of male mice. Alpha-Tubulin was used as the internal control. All experiments were repeated at least 3 times.

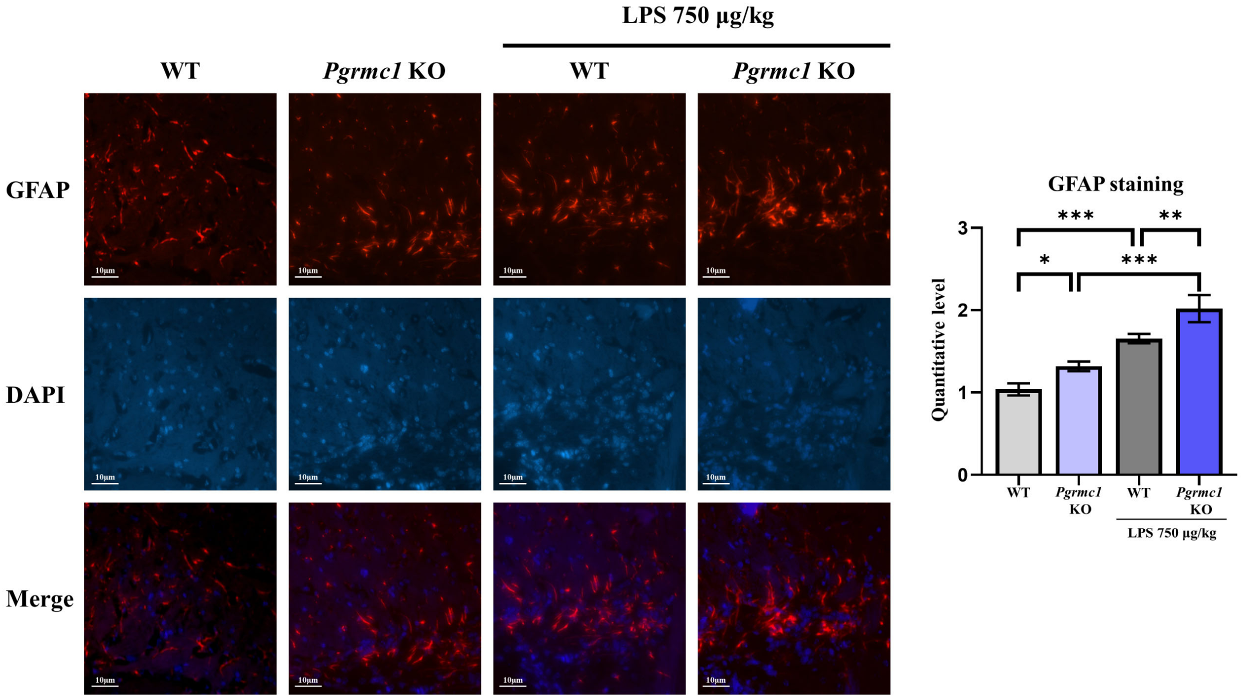

Figure S2. Loss of PGRMC1 increases the expression of glial fibrillary acidic protein (GFAP) in the mouse cerebrum. Astrocyte cells were characterized through GFAP staining (Scale bar, 10 µm). Representative immunohistochemistry (IHC) of cerebrum astrocytes in the mouse brain depicts GFAP (red) and DAPI (blue). Quantification of GFAP staining was analyzed using Image J, with white holes set as positive standards. Differences between means were assessed using one-way ANOVA, followed by Tukey's post-analysis. Values represent means +/- standard deviation. * $p < 0.05$, ** $p < 0.01$, *** $p < 0.001$ All experiments were replicated at least 3 times.

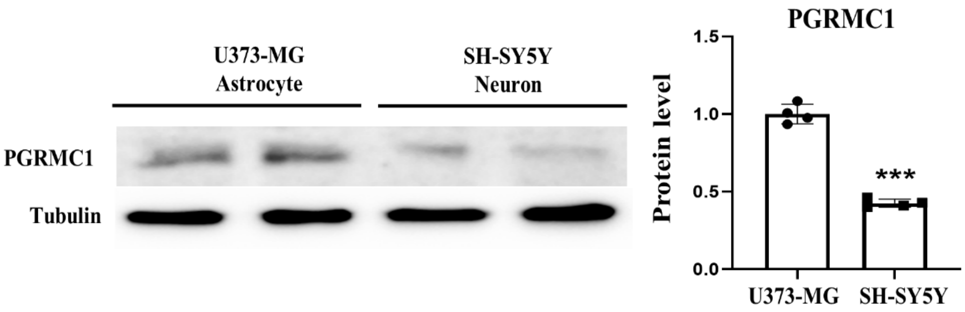

Figure S3. Expression of PGRMC1 in Astrocytes and Neuronal Cells. Western blot analysis and quantification of PGRMC1 protein levels were evaluated in U373-MG and SH-SY5Y. Alpha-tubulin was used as an internal control. Values represent means +/- standard deviation. *** $p < 0.001$. All experiments were repeated at least 3 times.

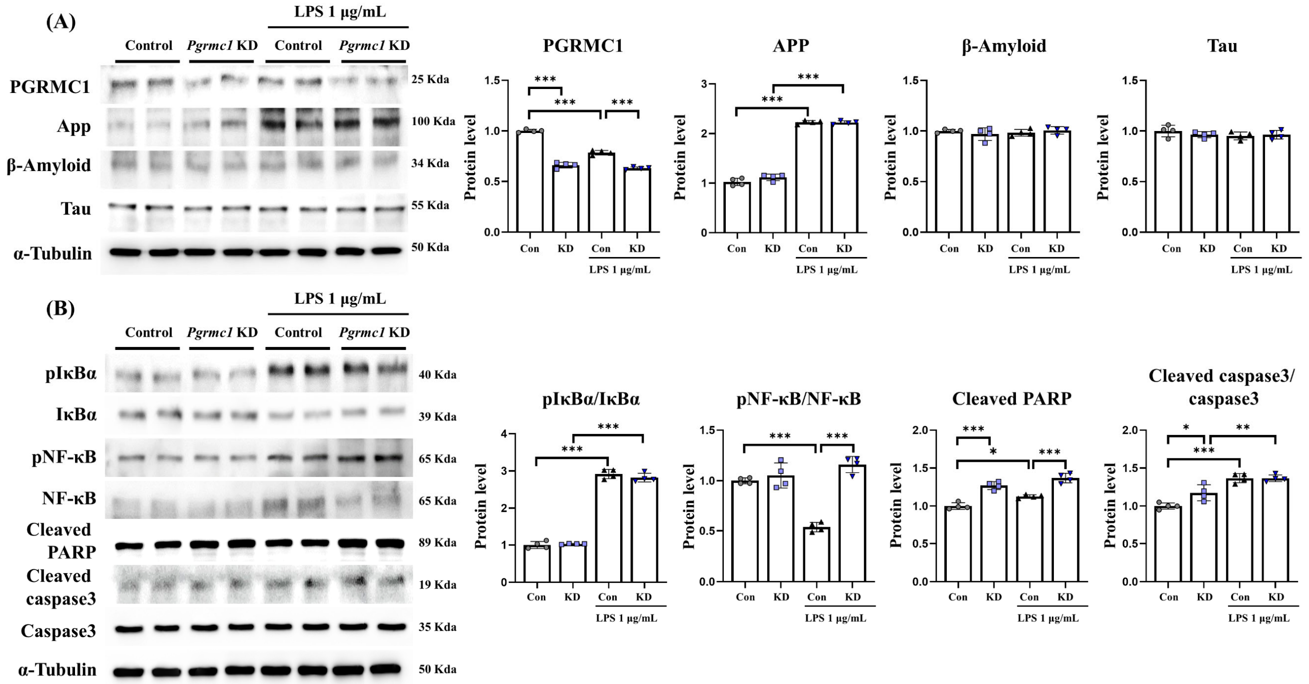

Figure S4. Downregulation of Pgrmc1 in SH-SY5Y cells has no effect on the levels of Alzheimer's disease and inflammation-related protein markers. (A) Western blot analysis and quantification of Alzheimer's disease-related protein were performed in SH-SY5Y cells. Alpha-Tubulin was used as an internal control. (B) Western blot analysis and quantification of inflammation-related protein were performed in SH-SY5Y cells. Alpha-Tubulin was used as an internal control. The differences between means were assessed through one-way ANOVA, followed by a Tukey post-analysis. Values represent means +/- standard deviation. * $p < 0.05$, ** $p < 0.01$, *** $p < 0.001$ ($n = 4$ in Control and KD groups, $n = 4$ in LPS-treated Control and KD groups). All experiments were repeated at least three times.

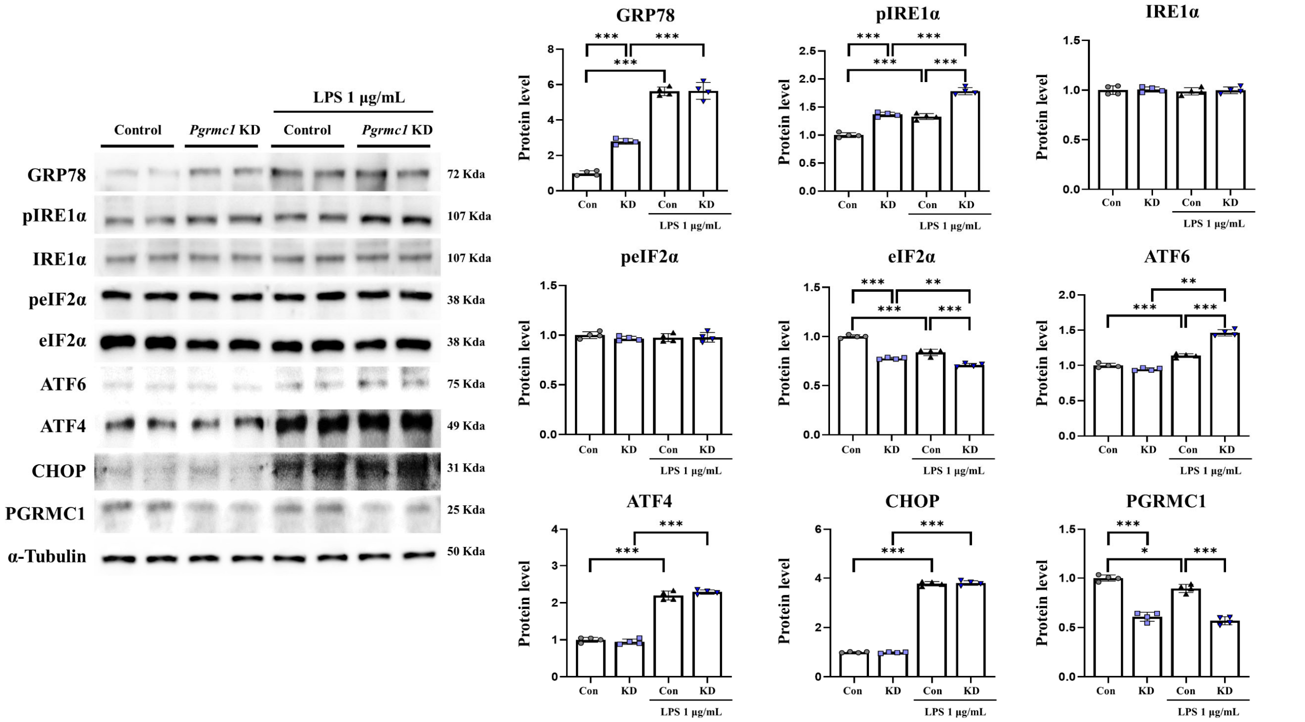

Figure S5. Downregulation of PGRMC1 in SH-SY5Y cells has negligible effects on the levels of ER stress-related protein markers. Western blot analysis and quantification of ER stress-related protein were performed in SH-SY5Y cells. Alpha-Tubulin served as the internal control. The differences between means were determined through one-way ANOVA, followed by Tukey post-analysis. Values represent means +/- standard deviation. * $p < 0.05$, ** $p < 0.01$, *** $p < 0.001$ ($n = 4$ in Control and KD groups, $n = 4$ in LPS-treated Control and KD groups). All experiments were repeated at least 3 times.
